# Real-Time Visual Tracking with Variational Structure Attention Network

**DOI:** 10.3390/s19224904

**Published:** 2019-11-09

**Authors:** Yeongbin Kim, Joongchol Shin, Hasil Park, Joonki Paik

**Affiliations:** Department of Image, Chung-Ang University, Seoul 06974, Korea; sawors2010@gmail.com (Y.K.); mbstel275@gmail.com (J.S.); hahaha2470@gmail.com (H.P.)

**Keywords:** visual tracking, convolutional neural network, variational auto-encoder, correlation filter

## Abstract

Online training framework based on discriminative correlation filters for visual tracking has recently shown significant improvement in both accuracy and speed. However, correlation filter-base discriminative approaches have a common problem of tracking performance degradation when the local structure of a target is distorted by the boundary effect problem. The shape distortion of the target is mainly caused by the circulant structure in the Fourier domain processing, and it makes the correlation filter learn distorted training samples. In this paper, we present a structure–attention network to preserve the target structure from the structure distortion caused by the boundary effect. More specifically, we adopt a variational auto-encoder as a structure–attention network to make various and representative target structures. We also proposed two denoising criteria using a novel reconstruction loss for variational auto-encoding framework to capture more robust structures even under the boundary condition. Through the proposed structure–attention framework, discriminative correlation filters can learn robust structure information of targets during online training with an enhanced discriminating performance and adaptability. Experimental results on major visual tracking benchmark datasets show that the proposed method produces a better or comparable performance compared with the state-of-the-art tracking methods with a real-time processing speed of more than 80 frames per second.

## 1. Introduction

Visual tracking is one of the most widely used computer vision algorithms. The goal of visual tracking is to estimate the position and scale of a specified target from the sequence of video frames. Among various conventional tracking algorithms, the discriminative correlation filter (DCF) approaches achieved an acceptable tracking performance using low-level hand-crafted features [1,2,3,4,5,6]. However, the lack of representation of hand-crafted features makes the tracking task inaccurate or even fail on challenging sequences.

Recently, with the advent of the large-scale datasets [7], the convolutional neural networks (CNNs) had great success in the visual tracking field. Since visual tracking requires rich representations, deep features extracted from pretrained CNNs models [8,9,10,11] are widely used to replace the hand-crafted features in the DCF framework. In particular, the tracking-by-detection based trackers [12,13,14] have exhibited unparalleled performance by combining detection and tracking in a unified framework. However, in contrast to DCF-based trackers, they require high computational load to target localization, and, as a result, it makes a real-time tracking impossible. To increase the processing speed for real-time tracking, Siamese networks have been recently proposed for visual tracking applications [15,16,17]. They were trained to compare the similarity between the initial and deformed target appearances. In particular, Valmadre et al. and Wang et al. proposed adaptive tracking approaches that pre-train the DCF in conjunction with the Siamese network while maintaining the major properties of the correlation filter [16,17]. However, an approximated solution of the DCF unavoidably results in a boundary effect because of the circulant structure of the Fourier transform, and the unbalanced weighting mechanism of the cosine window will aggravate the boundary effect, which results in the tracking performance degradation. To solve the negative effects, recent visual tracking approaches adopted the Siamese network-based DCF proposed by Valmadre and Wang, but they cannot guarantee a promising performance especially in real-time tracking applications.

**Figure 1:**
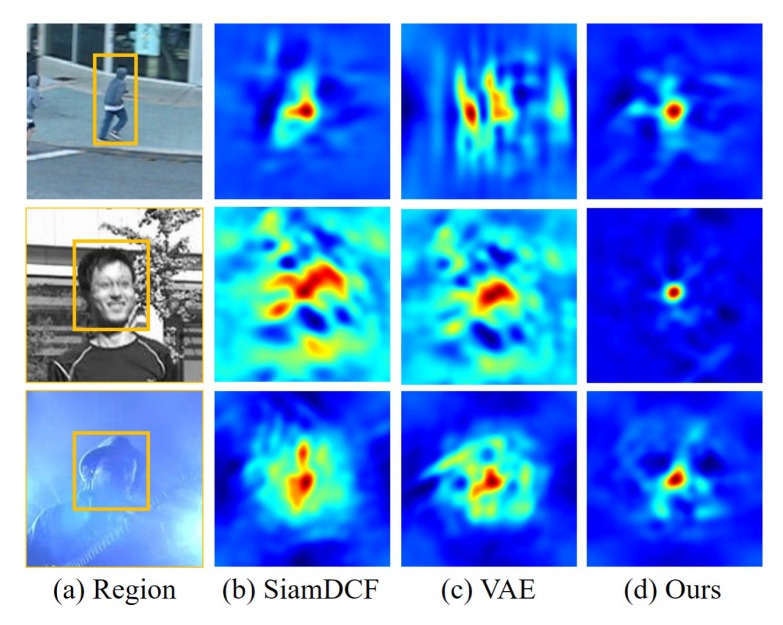
Response maps learned in different ways for correlation filters. (**a**) input images with a rectangular search region (*Jumping*, *Human4*, and *Shaking*, from top to bottom); (**b**) response maps of SiamDCF [17]; (**c**) response maps of our SiamDCF with a variational auto-encoder [18]; and (**d**) response maps of our method. Our method successfully removes the surrounding background clutters and focuses on the structure of the target, where the peak response value coincides with the true maximum correlation point.

To tackle this issue, we propose a fast and accurate tracking method using a structure–attention network to extract rich structures that are robust to the boundary effect problem. We first take SiamDCF [17] as a baseline Siamese tracking network for fast and adaptive tracking. To overcome the structure distortion problem caused by the boundary effect of the DCF, we train the DCF online through the proposed structure–attention network so that the DCF can learn the target structure that is robust to distortion. We use a variational auto-encoder as a structure–attention network to generate various and representative structures of the target. In addition, we train the structure–attention network by minimizing a novel reconstructed loss function combining two denoising criteria. The proposed two denoising criteria are designed based on two properties of DCF, cosine window weighting process, and shifted training samples by using a circulant matrix. Therefore, the denoising training process, by reflecting these properties in the denoising criteria enables the structure–attention network to capture robust features even in the boundary effect. In addition, the minimization of the proposed reconstructed loss, which represents the error of reconstructing both RGB input image and the corresponding feature map, allows the structure–attention network to generate a feature map without losing details of target structure, and can generate representative target structures. Figure 1 shows that our method can extract a robust structure of the target. The major contribution of the proposed work includes:

- We propose a structure–attention network to minimize the structure distortion due to boundary effect and to help learn the representative structure of target during the online training of correlation filter.
- We propose a novel reconstructed loss and two denoising criteria for training the structure–attention network. This allows for capturing robust structural features of the target even in the boundary effect without losing detailed information of the target.
- Experimental evaluations on various standard benchmark datasets demonstrate that our method achieves a better or comparable performances compared with the state-of-the-art tracking in accuracy and real-time tracking speed.

## 2. Related Works

Correlation filter-based approaches have played an important role in the visual tracking field because of their computational efficiency, accuracy, and robustness. Bolme et al. proposed a minimum output sum of squared error (MOSSE)-based correlation filter using single-channel features for real-time video tracking [1]. Henriques et al. proposed a kernelized correlation filter (KCF) using multi-channel features and circulant matrices [2,19]. Danelljan et al. used an adaptive color features in visual tracking for rich representation for the target [20]. To increase the accuracy in tracking a scale-variant object, Danelljan et al. proposed a scale estimation filter [3]. Choi et al. proposed a feature integration framework for visual tracking [4,5]. In addition to the multiple feature integration approaches, various algorithms were proposed to solve the intrinsic problems of correlation filters. Correlation filters often suffer from the boundary effect caused by cyclic-shift when training correlation filters. To overcome these issues, Galoogahi et al. proposed an alternating direction method of multipliers (ADMM) optimization for tracking [6], and Danelljan et al. proposed a spatial regularization method for correlation filters [21]. Chen et al. also proposed a new sparse model with a modulated template dictionary [22].

However, because of the common limitations in representing the target appearance using hand crafted features, convolutional features, which are extracted by CNNs pretrained on a large-scale dataset, such as ImageNet [7], have been widely used to improve the performance of correlation filter-based trackers [10,11,23]. Ma et al. adaptively trained correlation filters using hierarchical characteristics of pretrained CNN features [10]. Qi et al. adaptively integrate multi-correlation filter responses using an adaptive hedge algorithm [11]. Danelljan et al. integrated CNN features into [21] for performance improvement [23]. To overcome the drawback of single-resolution features, Danelljan et al. proposed an implicit interpolation method to integrate multi-resolution CNN features. Recently, a tracking-by-detection framework becomes one of the standard approaches for visual tracking. Different from correlation filter-based tracking methods, tracking is performed using a classifier which distinguishes target from background. Hong et al. proposed a framework to combine pretrained CNNs and used online SVMs to obtain target-specified saliency maps for tracking [24]. Instead of using a single classifier, Zhang et al. proposed a multi-expert restoration framework to address the drift-problem during tracking [25]. Nam et al. proposed a multi-domain learning framework for tracking [12]. This approach significantly improved the tracking performance. In spite of many attracting properties, most of tracking-by-detection frameworks commonly require high computational costs, and have limitation of features extracted by a pretrained CNN.

Recently, Siamese CNN architecture was used to compare the similarity of target through the end-to-end framework without any online fine-tuning [15,16,17]. These approaches are very successful and show remarkable performance improvement in real-time tracking. The biggest factor in the success of this approach is the use of pre-trained CNN models that are well-suited to tracking, rather than pre-trained CNN models on large-scale datasets. Bertinetto et al. proposed a fully convolutional Siamese tracking framework and introduced the correlation layers to estimate target positions [15]. Valmadre et al. improved the fully convolutional Siamese tracking framework by adding a correlation filter into the Siamese network, and achieved more shallow but efficient tracking [16]. Wang et al. proposed a similar Siamese network that can be trainable online by replacing the correlation layer by the discriminative correlation filter, and performed pre-training the Siamese network [17]. However, due to the boundary effect problem of correlation filters, it did not achieve a significant performance improvement compared with other Siamese network-based methods.

## 3. Proposed Method

**Figure 2:**
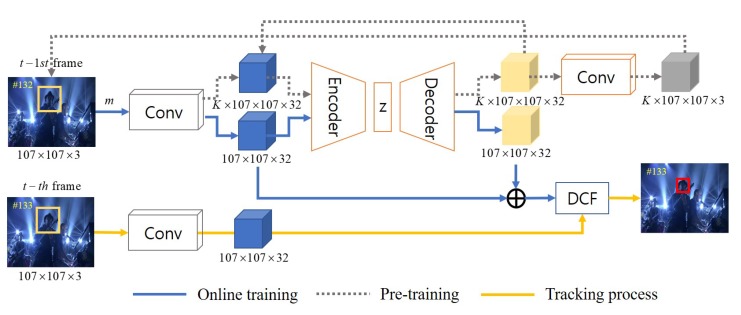
The overall network architecture and tracking process of the proposed tracking algorithm.

This section presents the proposed structure–attention network and online tracking process. Figure 2 shows the overall process of the proposed tracking algorithm.

### 3.1. Variational Auto-Encoder

Let *x* denote the data, *z* latent variable, and p(x|z) the distribution of generating data *x* given latent variable *z*. Since the inference of posterior p(z|x) is intractable to compute, the variational auto-encoder (VAE) utilizes q(z|x) to approximate the true posterior by optimizing the variational lower bound. The VAE maps the input data into latent variables q(z|x) via an encoder network, and then reconstructs p(x|z) from the latent variables via a decoder network. The variational lower bound, denoted as $L_{V}$, can be formulated as

(1) $$L_{V}=-D_{KL}(q_{\phi }\left(z|x_{i}\right)\left|\right|p_{\theta }\left(z\right))+E_{q_{\phi }\left(z|x_{i}\right)}\left[\log p_{\theta }\left(x_{i}|z\right)\right]$$

where the first term is the Kullback–Leibler divergence (KLD) of the approximated distribution from the true posterior, and the second term is expected reconstructed loss. Since the second term is not straightforward for the expected reconstructed term, we can reparameterize the *z* by using a differentiable transformation as [18]. We also assume that both q(z|x) and p(z) are Gaussian so that the KLD term can be analytically integrated. Hence, the standard VAE objective function can be formulated as

(2) $$L_{V}≃\frac{1}{2}\sum_{j=1}^{J}(1+\log\left(\sigma_{j}^{2}\right)-\mu_{j}^{2}-\sigma_{j}^{2})+\frac{1}{L}\sum_{l=1}^{L}\log p_{\theta }\left(x_{i}|z_{i,j}\right)$$

where *J* is the dimension of latent variable *z*, and {μ,σ} outputs of the deterministic encoder network. The reconstruction loss can be minimized using the cross entropy loss. More details can be found in [18].

### 3.2. Structure Attention Network

We propose to add a variational auto-encoder (VAE) sub-network in the upper Siamese path of SiamDCF as shown in Figure 2. The VAE is called a structure–attention network, which generates various and representative target structures. The encoder in the VAE subnet takes convolutional features of the previous (or t−1st) frame as input, and generates the latent vector *z*. More specifically, the encoder consists of three convolutional layers with batch-normalization and ReLU activation function followed by three fully-connected layers, and is considered as a nonlinear function of convolutional features $x\in R^{w\times h\times c}$ as

(3) $$\phi_{E}\left(x\right)=z\in R^{1\times m}$$

The decoder consists of three deconvolutional layers and one convolutional layer with the batch-normalization and ReLU activation function. The decoder takes the latent variable *z* as input, and generates both reconstructed feature map $y\in R^{w\times h\times c}$ and RGB image of size w×h×3 using another convolution layer. Table 1 shows the details of our structure–attention network.

### 3.3. Pre-Training

**Figure 3:**
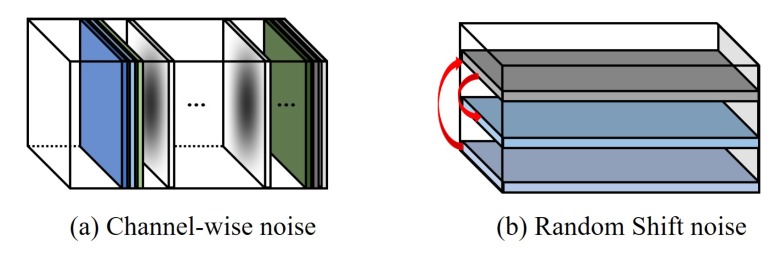
Proposed two structure noises: (**a**) channel-wise noise, which consists of randomly selected channels multiplied by the inverse–cosine window, and (**b**) random shift noise, which consists of randomly shuffled features.

In the pre-training step, the structure–attention network is pre-trained for the following two purposes: (i) capturing robust features even in the boundary effect problem, and (ii) generating various representative target features. To this end, we use dual-structure noises as denoising criteria as shown in Figure 3. The proposed dual-structure noises are based on the properties of the boundary effect problem, which is the intrinsic problem of the correlation filter.

The *channel-wise noise* consists of randomly selected channels multiplied by the inverse-cosine window. In the online tracking process, correlation filters can minimize background information by using cosine–window, and can accurately learn the target appearance. However, a center fitted weighting mechanism of cosine–window aggravates the boundary effect of the correlation filter, and makes the correlation filter learn unnecessary features. In this context, the structure–attention network is trained using an inverse–cosine window as a denoising criteria, to capture the robust features regardless of the center fitted weighting mechanism of cosine–window.

The *random shift noise* consists of randomly shifted rows and columns of feature vectors. Since the correlation filter is trained by using shifted training data using the circulant matrix, the structural information of the target is distorted, and therefore it is necessary to capture the robust features even in the shift. Thus, shifting feature vectors act like shifted training data during the training process of the correlation filter, and help the structure–attention network to capture robust features even with shifting.

**Figure 4:**
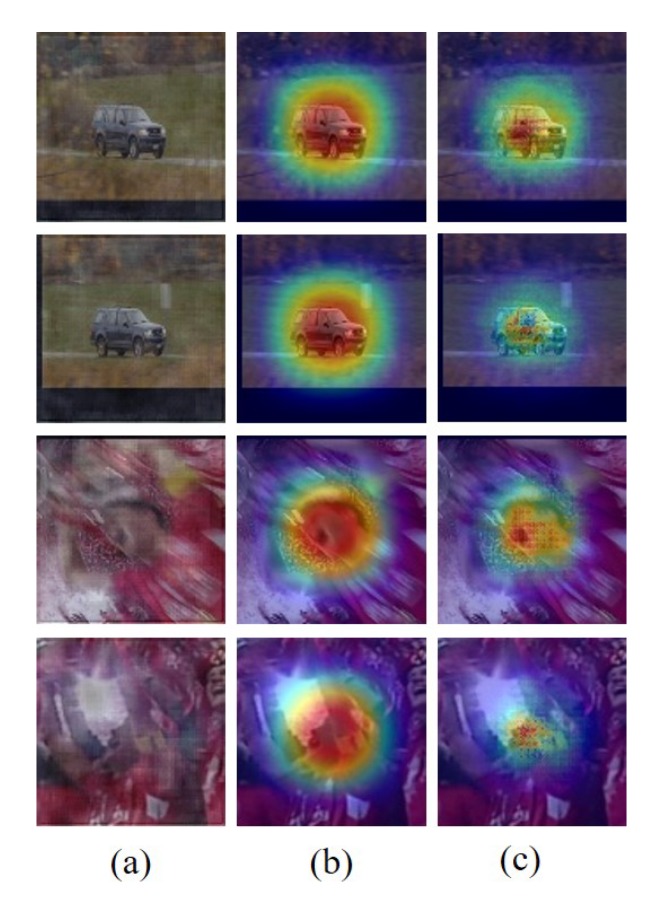
Visualization of the reconstructed attention maps (*Carscale*, and *Soccer*). (**a**) indicates an estimated target patch; (**b**) is a cosine window that is used to minimize background information; and (**c**) row is a visualized reconstructed attention maps through our structure–attention network. Unlike the unbalanced weighting mechanism of the cosine window that can capture the unnecessary background features, our structure–attention network can capture and highlight the robust target information.

**Figure 5:**
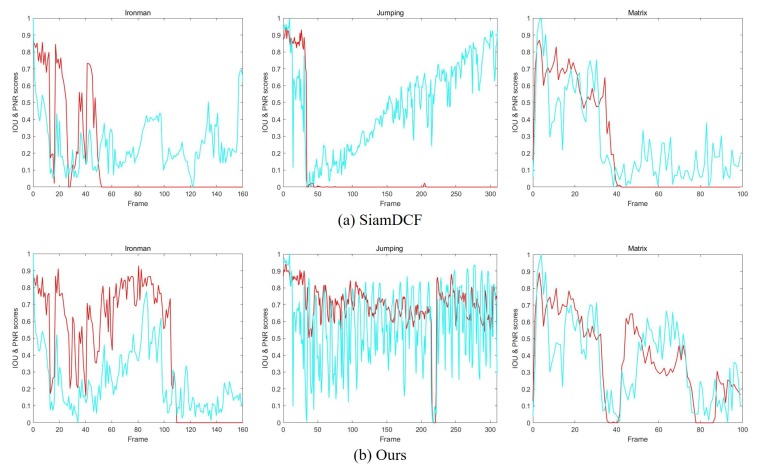
Visualization of the tracking result on three benchmark datasets including (*Ironman*, *Jumping*, and *Matrix*). (**a**) is a response graph of SiamDCF; and (**b**) is a response graph of ours. Red curves represent the IoU score of an estimated target and the ground-truth, while Green curves represent the normalized PNR value of the correlation response. Unlike the (**a**), the PNR graph of (**b**) is similar to that of IoU. This shows that the correlation filter can learn the robust features of the target online through the attention map generated from the structure–attention network.

To preserve the details of target structures, we propose a novel reconstructed loss function. The upper path of the Siamese network takes RGB images ${\left\{m_{i}\right\}}_{i=1}^{K}$ as input, and produces the convolutional feature map ${\left\{x_{i}\right\}}_{i=1}^{K}$ as output. The VAE takes the feature maps with a batch size *K* as input. Let ${\left\{{\tilde{x}}_{i}\right\}}_{i=1}^{K}$ denote the feature map which is corrupted by two noise structures. Given the latent variable *z*, different from the standard VAE, not only latent variable *z* but also input feature maps are corrupted, variational denoising reconstructed loss $L_{R}$ can be formulated as [26]:

(4) $$\begin{array}{cc} L_{D}= & E_{p({\tilde{x}}_{i}|x_{i})}E_{q(z|{\tilde{x}}_{i})}\left[\log\frac{p_{\theta }\left(x_{i},z\right)}{q_{\phi }\left(z|{\tilde{x}}_{i}\right)}\right]\\ ≃ & \frac{1}{D}\sum_{d=1}^{D}\left(\log\frac{p_{\phi }\left(x_{i},z_{d}\right)}{q_{\phi }\left(z_{d},{\tilde{x}}_{i}\right)}\right), \end{array}$$

where $p({\tilde{x}}_{i}|x_{i})$ represents the distribution of generating data given corrupted convolutional feature maps and latent variable. However, to design the VAE to reconstruct not only robust feature maps from the corrupted feature maps but also to preserve detail structural information of target, the original target information should be reflect to VAE. Hence, we can reformulate our reconstructed loss by adding image reconstruction term as:

(5) $$\begin{array}{cc} L_{R}= & L_{D}+E_{q_{\phi }\left(z|m_{i}\right)}\left[\log p_{\theta }\left(m_{i}|z\right)\right]\\ ≃ & \frac{1}{D}\sum_{d=1}^{D}\left(\log\frac{p_{\phi }\left(x_{i},z_{d}\right)}{q_{\phi }\left(z_{d},{\tilde{x}}_{i}\right)}+\log p_{\theta }\left(m_{i},z_{d}\right)\right), \end{array}$$

where $p({\tilde{x}}_{i}|x_{i})$ and $p(m_{i}|z)$ respectively represent the distribution of generating data given convolutional feature maps and latent variable. Different from the conventional denoising VAE criterion, we added an image reconstruction term in Equation (4). The second term makes the latent variable reflect the image structure to reconstruct feature maps, and allow reconstructed feature maps to preserve details of the target structure. To approximate the true posterior more stably, we can add the regularization term too. As a result, the objective function of our structure–attention network can be formulated as:

(6) $$\begin{array}{cc} L_{V}≃ & \frac{1}{2}\sum_{j=1}^{J}(1+\log\left(\sigma_{j}^{2}\right)-\mu_{j}^{2}-\sigma_{j}^{2})\\ \; & +\frac{1}{D}\sum_{d=1}^{D}\left(\log\frac{p_{\phi }\left(x_{i},z_{d}\right)}{q_{\phi }\left(z_{d},{\tilde{x}}_{i}\right)}+\log p_{\theta }\left(m_{i},z_{d}\right)\right), \end{array}$$

where *J* is the dimension of latent variable *z*, and μ and σ are outputs of variational parameter ϕ of the encoder network that takes corrupted feature maps ${\tilde{x}}_{i}$ as input. Figure 4 shows the reconstructed feature maps through the pre-trained structure–attention network. The reconstructed feature maps can attract attention to a representative and robust target structural features. Figure 5 also shows the tracking results using the proposed structure–attention network. We use the intersection-over-union (IoU) with the peak-versus-noise-ratio (PNR) which is introduced [27], to reveal the distribution of the correlation response map and to analyze the impacts of our attention map.

### 3.4. Online Tracking

Since the target appearance changes by frame, online training is required for adaptive tracking. The standard discriminative correlation filter based tracking method can be formulated as a ridge-regression problem:

(7) $$\min_{w}\left|\right|\sum_{i}w_{i}\cdot x_{i}-y|{|}^{2}+\lambda \sum_{i}\left|\right|w_{i}|{|}^{2}$$

where $x_{i}$ represents a set of feature maps of the training samples, and *y* is a desired output. The solution to obatin a desired correlation filter $w_{i}$ can be gained as:

(8) $${\hat{w}}_{i}=\frac{{\hat{x}}_{i}⊙{\hat{y}}^{*}}{{\hat{x}}_{i}⊙{\hat{x}}_{i}^{*}+\lambda }$$

where ∧ denotes the Fourier domain, * represents a complex conjugate, and ⊙ denotes the Hadamard product. Since the feature vectors are circulant matrix, computational load can be reduced. In order to prevent a distortion of structural information of the target due to the boundary effect during online training of the correlation filters, train the feature maps obtained through the structure–attention network together. From Equation (8), we can reformulate the correlation filter in online process as:

(9) $${\hat{w}}_{i}=\frac{\left({\hat{x}}_{i}+{\hat{s}}_{i}\right)⊙{\hat{y}}^{*}}{\left({\hat{x}}_{i}+{\hat{s}}_{i}\right)⊙\left({\hat{x}}_{i}^{*}+{\hat{s}}_{i}^{*}\right)+\lambda }$$

where *x* represents a feature map from the Siamese network, and *s* a structure feature map from the structure–attention network. The correlation filtering process in the *t*-th frame can be simplified as:

(10) $${\hat{w}}_{i}^{t}=\frac{\left(1-\eta \right)A_{i}^{t-1}+\eta \left({\hat{x}}_{i}^{t}+{\hat{s}}_{i}^{t}\right)⊙{\hat{y}}^{*}}{\left(1-\eta \right)B_{i}^{t-1}+\eta \left({\hat{x}}_{i}^{t}+{\hat{s}}_{i}^{t}\right)⊙\left({\hat{x}}_{i}^{t*}+{\hat{s}}_{i}^{t*}\right)+\lambda },$$

where *t* represents a frame index, η a online learning rate, and *A* and *B* respectively cross- and auto-correlations that are added to the structure–attention feature map.

## 4. Experimental Results

In this section, we introduce the details of our method, and evaluate our tracking algorithm on various benchmark datasets OTB2013 [28], OTB2015 [29], and Temple-Color-128 [30]. In particular, we evaluate the effectiveness of our structure–attention network through the multiple ablation study and detailed evaluation on various sequences. In addition, all experiment results can be found at [31].

### 4.1. Implementation Details

The Siamese network receives a 107×107×3 image as input. The structure–attention network receives a 107×107×32 feature map as input, and generates output of the same size. In the pre-training phase, we used Caltech-256 dataset [32] with batch size 64 with 50 epochs. We used Adam optimizer with learning rate 0.001. We implemented our algorithm in Python using the Pytorch library. In the online tracking phase, we set regularization parameter λ and online learning rate η to 0.0001 and 0.01, respectively. The proposed algorithm runs on a PC with an Intel Core i7 3.4 GHz CPU (Santa Clara, CA, USA), 32 GB RAM, and a Geforce GTX 1080 TI GPU (Santa Clara, CA, USA). In our settings, the average speed is 89 FPS.

### 4.2. Evaluation Methodology

We compare the performance of our tracking method with twelve state-of-the-art trackers including: SiamDCF [17], DSST [3], ACFN [5], SRDCF [21], SRDCFdecon [33], MEEM [34], Sturck [35], SiamFC [15], CFNet [16], ADNet-fast [36], CNN-SVM [24], and TRACA [37]. We follow the evaluation approaches introduced in the standard benchmark [28]. The performance of trackers is evaluated by using one-pass evaluation (OPE) with precision and success plots. The precision plots measure the percentage of frames where the distance between the estimated locations and the ground-truth is under a threshold. The success plots measure the overlap ratio between estimated bounding boxes and ground-truth. We set the distance threshold to 20 pixels in precision plots and use Area Under Curve (AUC) in success plots.

### 4.3. Evaluation on OTB2013

**Figure 6:**
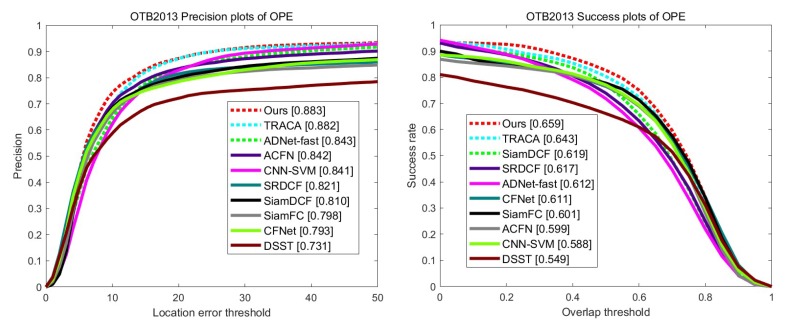
The precision and success plots on the OTB2013 [28] dataset by using one-pass evaluation.

We evaluate our tracking method on 50 video sequences using one-pass-evaluation with distance precision and overlap success ratio. Figure 6 shows both precision and success rate on the 50 video sequences. The proposed tracker achieved the state-of-the-art performance. Our method performs the best in both precision and success rate, and has a large margin in success rate compared with TRACA [37]. In particular, through our structure–attention network, our tracker outperforms a baseline Siamese tracker SiamDCF in a large margin.

### 4.4. Evaluation on OTB2015

**Figure 7:**
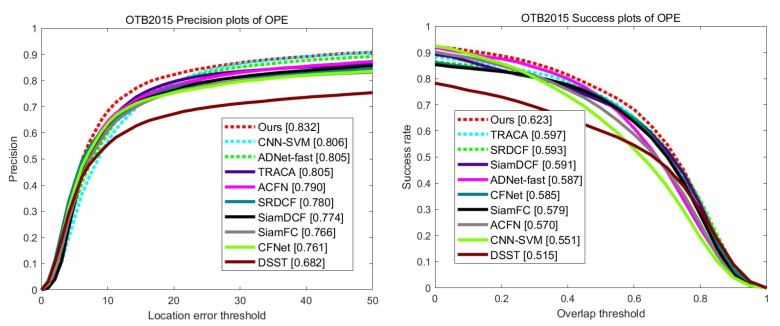
The precision and success plots on the OTB2015 [29] dataset.

We evaluate our algorithm on OTB2015 [29] dataset which contains more videos and hard datasets than OTB2013 [28]. This dataset includes 100 fully annotated video sequences. Figure 7 shows the overall results on OTB2015 dataset. Our method achieves the best result in both precision and success rate. In particular, compared to TRACA [37], which is ranked second by a small margin on OTB2013 dataset, our method outperforms with a large margin for both precision and success rate. This illustrates our method is more robust and accurate on challenging video sequences. In addition, the large margin between the proposed tracker and our baseline Siamese tracker SiamDCF [17] demonstrates that our structure–attention network can capture the robust structural features of target, and can train discriminative correlation filter adaptively even under the boundary effect problem.

**Figure 8:**
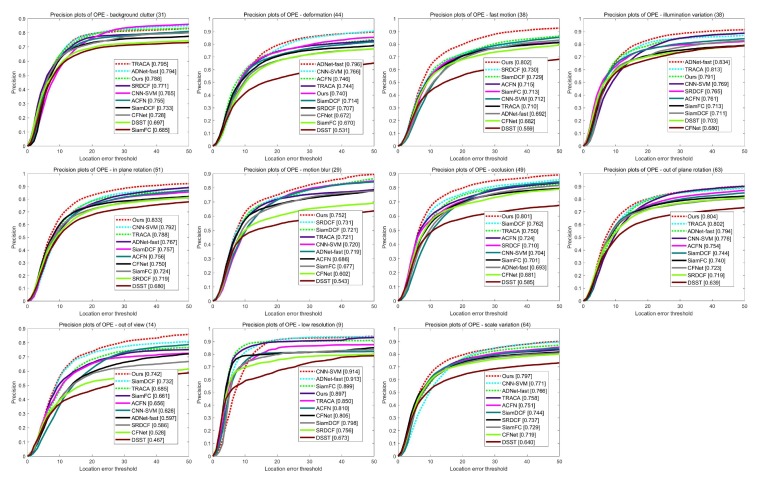
Comparison of precision plots over 11 attributes.

**Figure 9:**
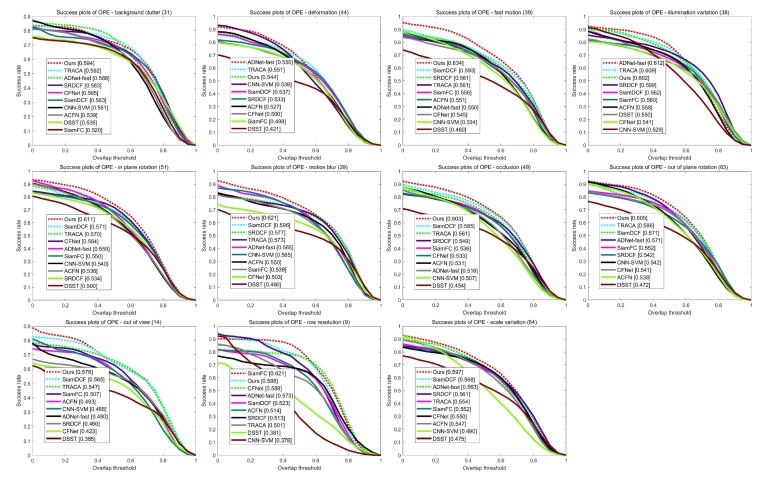
Comparison of success plots over 11 attributes.

**Figure 10:**
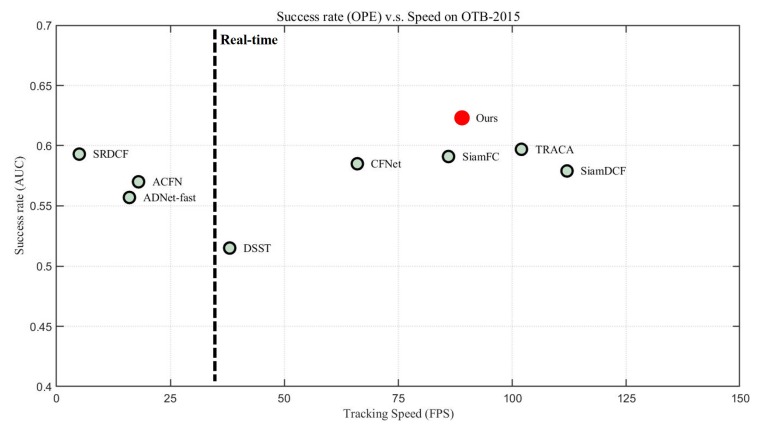
Comparison of accuracy vs. speed efficiency. Our tracker shows the best performance in accuracy, and sufficiently fast speed of over 80 fps.

Table 2 and Figure 8 illustrate the precision scores of 11 video attributes on the OTB2015 dataset. The proposed method shows the best performances in seven attributes. In addition, Table 3 and Figure 9 demonstrate the success rate scores of 11 video attributes. Our tracker achieves the best performances in eight attributes and the second best score in Low Resolution. This clearly shows the effectiveness of our structure–attention network. Figure 10 also shows that the proposed method outperforms other trackers in success rate versus speed.

### 4.5. Evaluation on TempleColor-128

**Figure 11:**
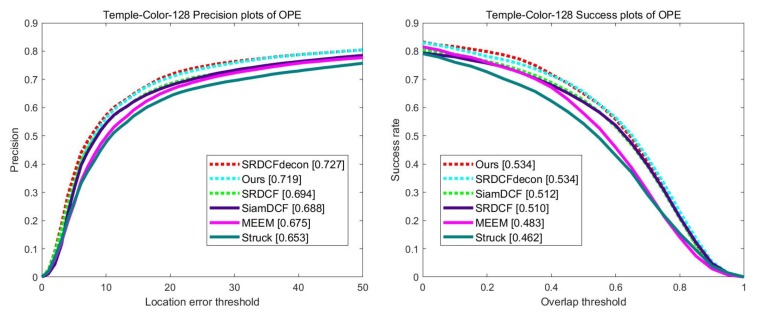
The precision and success rate overall sequences by using one-pass-evaluation on the TempleColor-128 dataset.

We compare our tracker on the TempleColor-128 [30] dataset containing 128 video sequences using one-pass-evaluation. Figure 11 illustrates both distance precision and overlap success rate on overall video sequences. Our tracker ranks second by a small margin on distance precision. However, while SRDCFdecon [33] has 1 fps of average speed, our tracker has over 89 fps in real time. Moreover, our tracker achieves the best result in overlap precision with SRDCFdecon. In particular, compared to our baseline tracker SiamDCF [17], our method outperforms SiamDCF with a large margin.

### 4.6. Ablation Study

**Figure 12:**
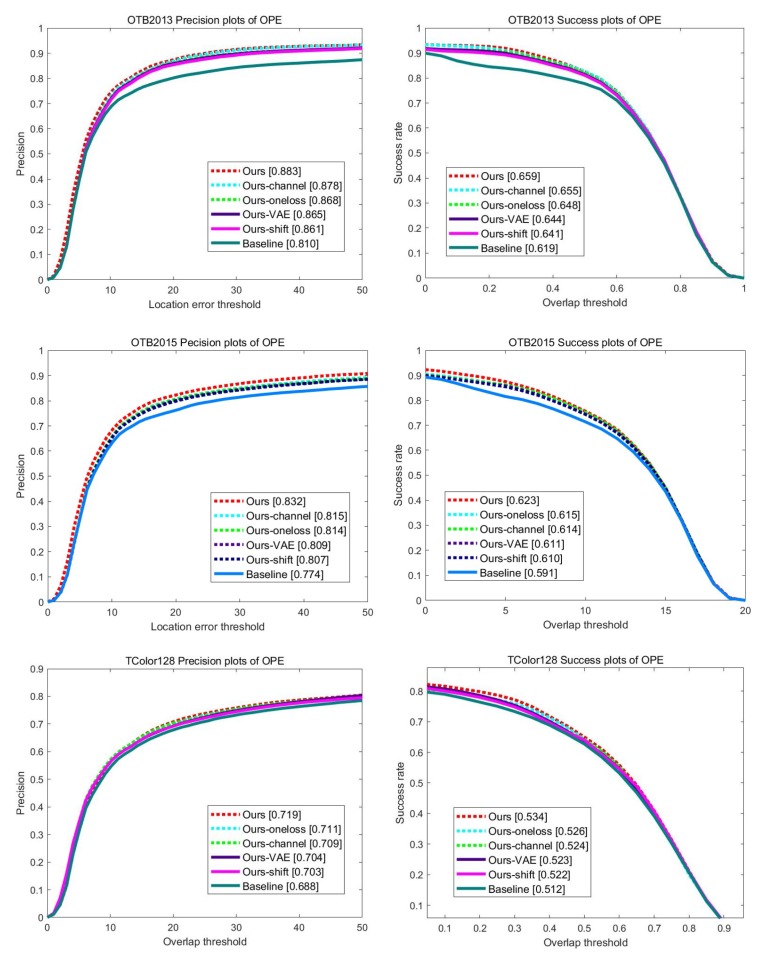
Internal comparison results of precision and success rate on OTB2013, OTB2015, and TColor128 datasets.

To analyze the impacts of the proposed method, we perform several ablation studies on OTB2013, OTB2015, and TColor128 datasets. We implement six variants of our tracker including: (i) *Baseline* is SiamDCF [17] which is our baseline Siamese tracking network, (ii) *Ours-shift* trains our structure–attention network using only shift noise, (iii) *Ours-channel* trains our structure–attention network using only channel-wise noise, (iv) *Ours-VAE* uses the standard variational auto-encoder (VAE) as structure–attention network, (v) *Ours-oneloss* trains the structure–attention network using only denoising reconstruction loss, and (vi) *Ours* is our complete model using both structure noises and the proposed reconstruction loss. Figure 12 shows the results on the overall datasets. Compared to SiamDCF, our complete model shows the best performances in both precision and success rate.

### 4.7. Qualitative Evaluation

**Figure 13:**
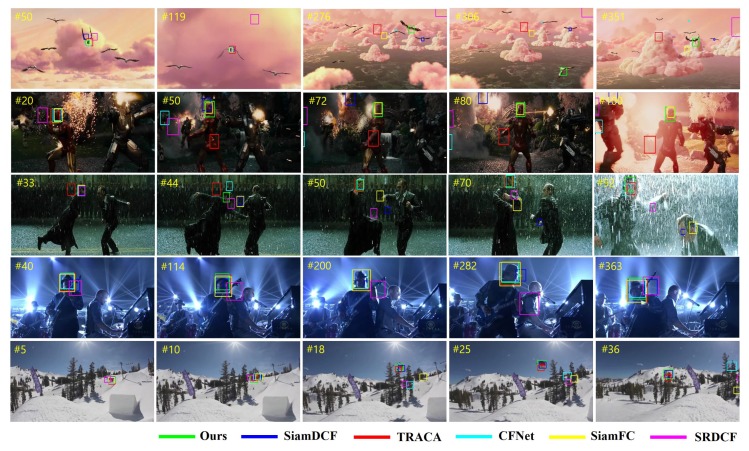
Qualitative comparison of our trackers with five trackers on OTB2015 dataset (from top to down are *Bird1*, *Ironman*, *Matrix*, *Shaking*, and *Skiing*). Our trackers achieve the best visual results with existing trackers in several challenging sequences.

We perform a qualitative evaluation of our method with five existing trackers including: SiamDCF, SiamFC, CFNet, TRACA, and SRDCF. Figure 13 illustrates several frames from five challenging sequences on OTB2015 dataset (*Bird1*, *Ironman*, *Matrix*, *Shaking*, and *Skiing*). In the *Bird1* and *Ironman* sequences, which are some of the most challenging sequences on the OTB2015 dataset, our tracker robustly tracks the target from the start to end frame even in the heavy occlusion and deformation. In the *Matrix* and *Skiing*, when the compared trackers struggling due to heavy scale variation and small size of target, our method can accurately estimate the scale of target. In particular, compared to our baseline tracker SiamDCF, our method shows the significant improvement in qualitative results. This proves the effectiveness of our structure–attention network which can robustly train the correlation filter even in the boundary effect problem.

## 5. Conclusions

In this paper, we presented a novel real-time tracking method based on the discriminative correlation filter with the proposed structure–attention network. To capture robust structural features even in the boundary effect problem of the correlation filter, our structure–attention network is trained with a novel reconstructed loss and dual structure noises. Using the structure–attention network, the correlation filter can learn representative and robust structural features. Extensive experiments on benchmark datasets have shown the effectiveness of our method.

## Figures and Tables

**Table 1:** The detail of each layer in the Structure Attention Network.

Layer	Input Channels	Output Channel	Filter Size	Stride	Padding
Conv1	32	64	4 × 4	2	1
BatchNorm-ReLU	64	64	-	-	-
Conv2	64	128	4 × 4	2	1
BatchNorm-ReLU	128	128	-	-
Conv3	128	64	4 × 4	2	1
BatchNorm-ReLU	64	64	-	-
Fc1	13 × 13 × 64	512	-	-	-
Fc2	13 × 13 × 64	512	-	-	-
Fc3	512	13 × 13 × 64	-	-	-
T-Conv1	64	128	4 × 4	2	1
BatchNorm-ReLU	128	128	-	-	-
T-Conv2	128	64	4 × 4	2	1
BatchNorm-ReLU	64	64	-	-	-
T-Conv3	64	32	4 × 4	2	1
BatchNorm-ReLU	32	32	-	-	-
Conv4	32	3	1 × 1	1	-
Tanh	3	3	-	-	-

**Table 2:** Distance precision (DP) scores of the 10 trackers in terms of different attributes: The top three results are shown in Red, Blue, and Green.

	Ours	CFNet	SRDCF	SiamFC	SiamDCF	ADNet	DSST	TRACA	CNN-SVM	ACFN
IV	0.791	0.680	0.765	0.713	0.711	0.834	0.703	0.813	0.761	0.761
SV	0.797	0.719	0.737	0.729	0.744	0.766	0.640	0.758	0.761	0.751
OCC	0.801	0.681	0.710	0.701	0.762	0.693	0.585	0.750	0.704	0.724
DEF	0.740	0.672	0.707	0.670	0.714	0.796	0.531	0.744	0.766	0.746
MB	0.752	0.602	0.731	0.677	0.721	0.719	0.543	0.720	0.720	0.686
FM	0.802	0.682	0.730	0.713	0.729	0.692	0.556	0.710	0.712	0.715
IPR	0.833	0.749	0.721	0.724	0.757	0.767	0.680	0.788	0.791	0.756
OPR	0.804	0.723	0.719	0.739	0.744	0.794	0.639	0.802	0.776	0.754
OV	0.742	0.528	0.586	0.661	0.732	0.597	0.467	0.685	0.626	0.656
BC	0.788	0.728	0.772	0.685	0.733	0.794	0.697	0.795	0.766	0.755
LR	0.897	0.805	0.757	0.899	0.798	0.913	0.673	0.850	0.914	0.810

**Table 3:** Average area under curve (AUC) scores of the 10 trackers in terms of different attributes: The top three results are shown in Red, Blue, and Green.

	Ours	CFNet	SRDCF	SiamFC	SiamDCF	ADNet	DSST	TRACA	CNN-SVM	ACFN
IV	0.602	0.541	0.599	0.560	0.562	0.612	0.550	0.608	0.529	0.558
SV	0.597	0.550	0.561	0.552	0.568	0.563	0.475	0.554	0.490	0.547
OCC	0.603	0.533	0.549	0.536	0.585	0.518	0.454	0.561	0.507	0.531
DEF	0.544	0.500	0.533	0.498	0.537	0.555	0.420	0.550	0.538	0.527
MB	0.621	0.503	0.577	0.539	0.596	0.565	0.460	0.573	0.565	0.550
FM	0.634	0.546	0.581	0.556	0.593	0.550	0.460	0.561	0.534	0.551
IPR	0.611	0.564	0.534	0.550	0.568	0.559	0.500	0.571	0.540	0.536
OPR	0.605	0.541	0.542	0.552	0.571	0.571	0.472	0.586	0.542	0.538
OV	0.576	0.423	0.460	0.507	0.565	0.479	0.385	0.547	0.488	0.493
BC	0.594	0.565	0.583	0.520	0.563	0.588	0.535	0.591	0.551	0.539
LR	0.598	0.588	0.513	0.621	0.523	0.573	0.381	0.501	0.378	0.514
